# Test-Retest Reliability of a Measure of Independence in Everyday Activities: The ADL Profile

**DOI:** 10.1155/2017/3014579

**Published:** 2017-01-22

**Authors:** Élisabeth Dutil, Carolina Bottari, Claudine Auger

**Affiliations:** ^1^School of Rehabilitation, Université de Montréal, Montreal, QC, Canada; ^2^Centre of Interdisciplinary Research in Rehabilitation of Greater Montreal (CRIR), Montreal, QC, Canada

## Abstract

**Background:**

Very few performance-based measures used in occupational therapy have established test-retest reliability coefficients.

**Objectives of Study:**

This study presents the test-retest reliability of the task and operation scores of a performance-based measure of independence in everyday activities called the ADL Profile.

**Methods:**

20 adults with severe traumatic brain injury (mean age 28.4 years; SD 9.9) were tested on two occasions with the 17 tasks (personal care, home, and community) of the ADL Profile. Kappa coefficients were calculated on both task and operation scores (formulating goal, planning, executing, and goal attainment).

**Findings:**

Test-retest reliability was moderate to almost perfect on task and operation scores of all 17 tasks. The three tasks with only moderate agreement were more novel and complex (e.g., making a budget) for the participants.

**Relevance to Clinical Practice:**

Use of measures that are stable over time is essential for treatment planning and research. Repeat testing is crucial with clients that require long periods of treatment (acute care, rehabilitation, and community integration) and multiple measurements of ADL independence.

**Limitations:**

The small sample size is a limit of the study.

**Recommendations for Further Research:**

Alternate versions of the three tasks with only moderate agreement would need to be developed and other psychometric properties established.

## 1. Introduction

Documenting the effect of treatment interventions requires the use of stable measures to ensure that changes pre-post intervention in client functioning deemed to be a result of specific treatments are not a measurement error caused by an unstable tool. Most frequently used outcome measures in rehabilitation are generally measures of everyday activities. Much literature has shown the importance of considering the impact of executive functions on independence in everyday activities and the need for such measures to be administered in a real-world context [1]. For these measures to be sensitive to the impact of executive function deficits on independence, the latter must include elements of novelty and complexity [2]. The challenge of using such measures on two separate occasions with the same individual, such as when tests are administered before and after an intervention, is that evaluation measures are no longer novel on the second administration of the test, directly impacting its stability [3]. Hence, unstable tests can lead to erroneous positive treatment effect conclusions as the person is found to improve on the test without any assurance that improvements are not related to the person having learned the test rather than them having fundamentally improved.

A recent study by Poulin et al. [4] showed that very few performance-based measures of executive functions used in occupational therapy with a stroke population have established test-retest reliability coefficients. In fact, out of 19 tests examined, only two, the Assessment of Motor and Process Skills [5, 6] and the Virtual Environment Technology based cognitive assessment program [7], had demonstrated test-retest reliability. However, test-retest reliability study of the Assessment of Motor and Process Skills with a traumatic brain injury population was completed on only two of the numerous tasks included within this test and the delay between the two administration instances was a single day, thus weakening the findings of the study [8].

The present study examined the test-retest reliability of the task and operation scores of a performance-based measure of independence in everyday activities called the Activities of Daily Living (ADL) Profile [9]. This measure was specifically developed to consider the impact of executive deficits on independence in everyday activities, with a particular emphasis having been given to the consideration of such fundamental abilities as goal formulation and planning. The ADL Profile was developed with the intent of offering occupational therapists a measure that would guide the elaboration of therapeutic objectives and monitor longitudinal change. It is meant to be first administered in acute care where the focus is more on personal Activities of Daily Living, then in rehabilitation, and, finally, in the community. Hence, as the test can and should be repeated over time to evaluate changes in performance, documenting the test-retest reliability of the test is of utmost importance.

## 2. Methods

### 2.1. Subjects

The target population were persons who had sustained a severe traumatic brain injury (TBI). Participants were actively receiving rehabilitation services, either inpatient or outpatient, at the time of the study. Inclusion criteria were as follows: patient age between 16 and 65 years, severe TBI within the past two years, and proficiency in French. The severity of the TBI was estimated with the Glasgow Coma Scale (GCS) (GCS score ≤ 8 = severe) and duration of posttraumatic amnesia (several weeks), as measured by the Galveston Orientation and Amnesia Test (GOAT). Persons with a history of a psychiatric disorder documented in the medical chart were excluded.

Sample size decisions were based on the feasibility of evaluating clients twice in their own home and community environment or in certain instances in an acute care or rehabilitation setting, with a performance-based measurement tool that involved the observation of 17 personal, domestic, and community tasks. A sample size of 20 subjects was deemed feasible for the current study considering the time and cost involved in administering this type of test. The Institutional Review Board of the research center approved the study and informed consent was obtained according to the legal ability of participants (direct consent or through a legal representative).

### 2.2. Instrument

The ADL Profile [9] was developed based on the theoretical framework of the Model of Cerebral Functioning [10]. The Model of Cerebral Functioning and its presentation of a unit of cognitive functioning dedicated to goal formulation, planning, and error detection and correction associated largely to the frontal lobe highlighted the need for the test to use a minimally structured approach in which therapists allow clients to formulate goals and plan the tasks they will carry out during a specific assessment period in which the evaluator observes the person's performance. This minimally structured approach is the originality of the test as no other measure developed and validated to date has been shown to be as unstructured for the client or as representative of real-life functioning as the ADL Profile. Participant performance is scored on each of the following components: ability to formulate goals, to develop strategies or plans to attain these goals, to apply and adjust them during the execution, and to evaluate and correct the results in relation to the goals [11]. The test is administered in the person's environment. This would ideally be the person's own home and community environment, though certain activities can also be tested within an acute care hospital, a rehabilitation hospital, or a long-term care hospital.

The concept of ADL in the ADL Profile is divided into three dimensions, based on Lawton's Environmental Model: personal care, home, and community activities [12]. A number of tasks (17) were identified as representative of these environments. These include such tasks as bathing and putting on clothes within the personal domain, preparing a hot meal and doing laundry in the home domain and using public transportation, telephoning for information, paying a bill, and making a budget for the community domain. Therapists observe the person completing each of these tasks and note observable behaviours as well as verbalisations that inform the therapist of how the person is thinking through the task that must be realized. Task-related observations are then grouped into 4 operations (formulating a goal, planning, carrying out the task, and verifying attainment of the goal) to facilitate a more precise identification of specific areas of breakdown in task performance. The scoring procedure then takes into consideration the degree of independence of the person when he or she carries out each of the various tasks (task score) and the manner in which he or she carries them out (operations). The scoring scales for operations and tasks make it possible to determine the presence or absence of difficulties with execution and the type of assistance required from the therapist to complete the components of each operation (verbal, physical, or both). For each observed operation and each task, the evaluator gives a score according to a four-point ordinal scale of independence (3: independence; 2: independence with difficulty; 1: requiring verbal, physical, or verbal and physical assistance; and 0: being unable to complete the task despite the assistance offered by the examiner). The lowest of these operation scores is used to determine the task score. Tool development [9] and interrater reliability with a severe TBI population were previously described [13, 14]. Interrater reliability, based on four trained occupational therapy raters, individually scoring the videos of 19 subjects without any consultation between raters, shows kappa statistics ranging between 0.23 (acceptable reliability) and 0.72 (substantial reliability).

### 2.3. Procedure

All participants were tested twice with all 17 tasks of the performance-based ADL Profile assessment within their home and community environment for some tasks and within the rehabilitation center for other tasks. They were tested on both occasions by one of four occupational therapists trained to administer the ADL Profile and experienced in testing TBI subjects. The same occupational therapist always administered the test on both occasions to any one participant. The target time for the second administration of the test was between one and two weeks following the first administration of the test. This time period was selected to minimize the effect of possible confounding variables such as recovery and learning effects, which could affect the data. Some studies have shown that though the optimal time-interval between testing will vary depending on the construct being measured, on the stability of the construct over time and on the target population, the target time of 2 weeks is the most frequently recommended interval [15].

### 2.4. Data Analysis

The statistical analysis was performed using Cohen's kappa statistic, the statistic that is recommended for test-retest studies of ordinal scales [16]. The kappa statistic measures interrater agreement, a more robust measure than percent agreement due to its consideration of agreement occurring by chance alone. Kappa statistics vary between 0 and 1 with values of 1 representing perfect agreement and values of 0 representing complete chance agreement. The Landis and Koch scale [17] is the standard to qualify the degree of agreement. This scale is interpreted as follows: almost perfect (0.81–1.00), substantial (0.61–0.8), moderate (0.41–0.6), acceptable (0.21–0.4), fair (0–0.2), and poor (less than 0).

## 3. Results

### 3.1. Participants

Twenty individuals having sustained a severe TBI participated in the study. The average age of the sample was 28.4 years [standard deviation (SD) 9.9]. 80% (*n* = 16) were males, 65% (*n* = 13) were single at the time of injury, 72.2% (*n* = 14) had completed their high school education, and 80% (*n* = 16) were working prior to their injury. Ninety-five percent (*n* = 19) of participants had been injured in a motor vehicle accident. The average Glasgow Coma Scale score at admission was 5.7 (SD 1.3) and, at day 2, the average Glasgow Coma Scale score was 7.4 (SD 1.7). Coma duration lasted an average of 49.5 days (SD 63.1) and average duration of posttraumatic amnesia was 107.8 days (SD 141.8). Time since injury varied from 3 months after injury to 2 years and over, 90% (*n* = 18) of whom were tested 6 months or more after injury, while the remaining two participants were tested at 3 months after injury. Fifty percent (*n* = 10) were living at home at the time of the study, 45% (*n* = 9) were receiving inpatient services in rehabilitation settings, and 5% (*n* = 1) were living in transition homes. The average interval between both tests was 22.9 days with a standard deviation of 12.7 days.

### 3.2. Agreement between Task Scores

Kappa values for all task scores are presented in Table 1. The results showed that test-retest reliability for task scores ranged from moderate (kappa = 0.45) to almost perfect (kappa = 0.93). Overall, the highest agreement was observed in the personal care domain as 20% of task scores in this domain had almost perfect agreement and 80% had substantial agreement. All of the task scores in the home domain had substantial agreement. The lowest agreement was in the community domain where 28.5% of task scores had almost perfect agreement, 28.5% had substantial agreement, and 42.8% had moderate agreement. A closer examination of the average scores at times 1 and 2 for 2 tasks that had moderate agreement (i.e., telephoning for information and making a budget) reveals a slightly increased average score at time 2.

### 3.3. Agreement between Operation Scores

Kappa values for each of the 4 operations are presented in Tables 2–5. Reliability for goal formulation varied between moderate (kappa = 0.59) and almost perfect (kappa = 0.90). Reliability for goal formulation was highest for tasks in the personal domain and variation was highest for community activities.

Reliability for planning varied between moderate (kappa = 0.42) and almost perfect (kappa = 1.00), with reliability being overall stronger than that for the goal formulation operation. Reliability on this operation was weakest for preparing a hot meal and making a budget.

Reliability for the “carrying out” operation varied between moderate (kappa = 0.45) and almost perfect (kappa = 0.87), with reliability being weakest for the tasks of preparing a light meal and making a budget.

Reliability for the “verifying attainment of the initial goal” operation varied between moderate (kappa = 0.39) and almost perfect (kappa = 0.86), with reliability being weakest for the task of making a budget.

In conclusion, when we consider the kappa coefficients of all 4 operations and task scores together the reliability was found to be the weakest for the task of making a budget, followed by the tasks of calling for information and shopping.

## 4. Discussion

From the above results, it appears that the present evaluation of the ADL Profile has overall substantial to almost perfect reliability when one rater repeatedly scores the tasks on the same participants in the same environment.

The reliability coefficients of the test-retest study were high, thus reflecting stability over repeated measures. Three tasks had only moderate agreement (telephoning for information, using an automatic teller, and making a budget), the lowest level of agreement in this study. This lowest level of agreement can be explained by the possibility of memory and learning effect by the subject or the fact that the task was more familiar to the participants and thus less challenging on second test administration. In fact, in terms of the effect of memory, the rater observed that some participants used strategies to remember the steps of the tasks (e.g., keeping a paper with the phone number that had been found in the first search of the task requiring that the participant calls a bus company to obtain a bus schedule), transforming their level of independence on the task to a greater level of independence on second administration of the test. As these tasks are among the least familiar tasks to the participants and the more complex tasks within the ADL Profile tool, participants overall were more dependent on these tasks on the first administration of the test and more likely to perform better on the second administration as the tasks were then less novel and complex for them. This same problem has been highlighted numerous times in the literature examining the test-retest reliability of measures of executive functions [3]. It is well known that a test is only novel once and that a second administration of such tests has a decreased ability to identify deficits in executive functions. What has thus been recommended in the literature is to have alternate versions of the tasks to maintain the novelty element of the test. This would need to be considered as a future avenue of research for these 3 specific tasks within the ADL Profile. Another approach to resolving this situation would be to revise the protocol of the tool to verify whether changes can be made to the protocol to limit the influence of such variables as memory and learning effect. However, it remains that our results are overall quite positive as 14 of the 17 tasks of the ADL Profile had overall high stability, highlighting the value of using a test with more than two or three tasks that may limit the learning effect as the tasks are too numerous to be remembered between the two test administration instances.

Finally, results of the test-retest study showed higher stability of the measure over time than interrater reliability, for most task scores. Two task scores (telephoning for information and using an automatic teller) showed slightly weaker test-retest agreement than interrater reliability. Performance on both of these tasks is thus more likely, than other tasks of the ADL Profile, to improve on second administration of the test, likely due to their decreased novelty on second administration.

*Study Limits*. Although the sample size was comparable to numerous other test-retest reliability studies [18], small sample sizes have the limit of creating some instability in the kappa coefficients and results must be interpreted with caution. However, to have obtained such high test-retest reliability coefficients with a heterogeneous sample of severe TBI, having been tested in varied environments (home and rehabilitation settings) at an average of a three-week interval between tests, shows the generalizability strength of the ADL Profile and its pertinence for clinical use.

## 5. Conclusion

The ADL Profile demonstrates overall substantial to almost perfect test-retest reliability, particularly pertaining to task scores, for its intended use with persons with a TBI. It serves as a framework by which occupational therapists can observe the person carrying out a series of activities in a real-world environment and categorize their observations into a formal assessment that can be used to guide their clinical interventions. Few other studies have reported the test-retest reliability of performance-based measures administered in the person's home and community environment. Further research is required to investigate its sensitivity to detect change and its ability to predict social participation.

It is hoped that this tool will yield the pertinent information necessary to adopt better rehabilitation strategies. This should result in more successful social and vocational reintegration for these persons.

## Figures and Tables

**Table 1:** Test-retest reliability of the task scores of the ADL Profile (*N* = 20).

Tasks	Session 1		Session 2		Kappa	SD	Landis and Koch scale
	$\overset{-}{X}$	S.D.	$\overset{-}{X}$	SD			
*Personal*							
(1) Bathing	1.60	1.32	1.42	1.26	0.79	0.10	Substantial
(2) Grooming	1.40	1.19	1.47	1.31	0.73	0.11	Substantial
(3) Toileting	1.80	1.36	1.85	1.42	0.75	0.12	Substantial
(4) Putting on clothes and shoes	1.50	1.28	1.63	1.42	0.65	0.12	Substantial
(5) Having a meal	1.80	1.28	1.85	1.31	0.93	0.07	Almost perfect

*Home*							
(6) Preparing a light meal	1.45	1.15	1.58	1.30	0.74	0.11	Substantial
(7) Preparing a hot meal	1.00	1.11	1.06	1.16	0.66	0.13	Substantial
(8) Doing daily house cleaning	1.35	1.18	1.47	1.35	0.73	0.11	Substantial
(9) Doing weekly house cleaning	1.10	1.10	1.06	1.16	0.66	0.12	Substantial
(10) Doing laundry	1.00	1.08	0.94	1.14	0.67	0.12	Substantial

*Community*							
(11) Walking or moving outdoors	1.35	1.27	1.55	1.43	0.64	0.12	Substantial
(12) Using public transportation	1.20	1.20	1.25	1.21	0.85	0.10	Almost perfect
(13) Shopping	1.35	1.22	1.30	1.17	0.73	0.12	Substantial
(14) Telephoning for information	0.94	1.30	1.00	1.28	0.45	0.13	Moderate
(15) Paying a bill	0.60	0.68	2.38	1.04	0.86	0.09	Almost perfect
(16) Using an automated banking machine	1.42	1.39	1.39	1.42	0.53	0.14	Moderate
(17) Making a budget	0.70	0.86	0.90	1.02	0.53	0.14	Moderate

**Table 2:** Test-retest reliability of the operation of formulating a goal of the ADL Profile.

Tasks	Kappa	SD	Landis and Koch scale
*Personal*			
(1) Bathing	0.79	0.11	Substantial
(2) Grooming	0.79	0.11	Substantial
(3) Toileting	0.78	0.11	Substantial
(4) Putting on clothes and shoes	0.70	0.12	Substantial
(5) Having a meal	0.90	0.09	Almost perfect

*Home*			
(6) Preparing a light meal	0.79	0.11	Substantial
(7) Preparing a hot meal	0.71	0.12	Substantial
(8) Doing daily house cleaning	0.77	0.12	Substantial
(9) Doing weekly house cleaning	0.67	0.14	Substantial
(10) Doing laundry	0.76	0.12	Substantial

*COMMUNITY*			
(11) Walking or moving outdoors	0.84	0.10	Almost perfect
(12) Using public transportation	0.68	0.12	Substantial
(13) Shopping	0.59	0.13	Moderate
(14) Telephoning for information^*∗*^			
(15) Paying a bill^*∗*^			
(16) Using an automated banking machine	0.60	0.12	Moderate
(17) Making a budget^*∗*^			

^*∗*^The examiner formulates the goals for these tasks. Thus, no scores are given.

**Table 3:** Test-retest reliability of the operation of planning of the ADL Profile.

Tasks	Kappa	SD	Landis and Koch scale
*Personal*			
(1) Bathing	0.79	0.11	Substantial
(2) Grooming	0.93	0.07	Almost perfect
(3) Toileting	0.65	0.12	Substantial
(4) Putting on clothes and shoes	0.84	0.11	Almost perfect
(5) Having a meal	1.00	0.00	Almost perfect

*Home*			
(6) Preparing a light meal	0.92	0.08	Almost perfect
(7) Preparing a hot meal	0.56	0.14	Moderate
(8) Doing daily house cleaning	0.76	0.13	Substantial
(9) Doing weekly house cleaning	0.75	0.13	Substantial
(10) Doing laundry	0.77	0.12	Substantial

*Community*			
(11) Walking or moving outdoors	0.92	0.08	Almost perfect
(12) Using public transportation	0.92	0.08	Almost perfect
(13) Shopping	0.60	0.12	Moderate
(14) Telephoning for information	0.84	0.10	Almost perfect
(15) Paying a bill	0.85	0.09	Almost perfect
(16) Using an automated banking machine	0.92	0.07	Almost perfect
(17) Making a budget	0.42	0.14	Moderate

**Table 4:** Test-retest reliability of the operation of carrying out the task of the ADL Profile.

Tasks	Kappa	SD	Landis and Koch scale
*Personal*			
(1) Bathing	0.73	0.12	Substantial
(2) Grooming	0.87	0.09	Almost perfect
(3) Toileting	0.72	0.11	Substantial
(4) Putting on clothes and shoes	0.67	0.12	Substantial
(5) Having a meal	0.86	0.09	Almost perfect

*Home*			
(6) Preparing a light meal	0.58	0.14	Moderate
(7) Preparing a hot meal	0.65	0.12	Substantial
(8) Doing daily house cleaning	0.65	0.13	Substantial
(9) Doing weekly house cleaning	0.71	0.12	Substantial
(10) Doing laundry	0.72	0.12	Substantial

*Community*			
(11) Walking or moving outdoors	0.73	0.11	Substantial
(12) Using public transportation	0.77	0.11	Substantial
(13) Shopping	0.73	0.12	Substantial
(14) Telephoning for information	0.72	0.12	Substantial
(15) Paying a bill	0.87	0.08	Almost perfect
(16) Using an automated banking machine	0.61	0.13	Substantial
(17) Making a budget	0.45	0.13	Moderate

**Table 5:** Test-retest reliability of the operation of verifying the attainment of the initial goal.

Tasks	Kappa	SD	Landis and Koch scale
*Personal*			
(1) Bathing	0.86	0.09	Almost perfect
(2) Grooming	0.80	0.10	Substantial
(3) Toileting	0.72	0.11	Substantial
(4) Putting on clothes and shoes	0.64	0.13	Substantial
(5) Having a meal	0.82	0.11	Almost perfect

*Home*			
(6) Preparing a light meal	0.70	0.13	Substantial
(7) Preparing a hot meal	0.78	0.11	Substantial
(8) Doing daily house cleaning	0.70	0.12	Substantial
(9) Doing weekly house cleaning	0.54	0.14	Moderate
(10) Doing laundry	0.72	0.12	Substantial

*Community*			
(11) Walking or moving outdoors	0.78	0.11	Substantial
(12) Using public transportation	0.76	0.12	Substantial
(13) Shopping	0.59	0.13	Moderate
(14) Telephoning for information	0.51	0.14	Moderate
(15) Paying a bill	0.86	0.09	Almost perfect
(16) Using an automated banking machine	0.79	0.10	Substantial
(17) Making a budget	0.39	0.13	Acceptable
